# Effectiveness of a physiotherapist delivered cognitive-behavioral patient education for patients who undergoes operation for total knee arthroplasty: a protocol of a randomized controlled trial

**DOI:** 10.1186/s12891-017-1476-6

**Published:** 2017-03-21

**Authors:** Sara Birch, Maiken Stilling, Inger Mechlenburg, Torben Bæk Hansen

**Affiliations:** 1 Department of Physiotherapy and Occupational therapy, Holstebro Regional Hospital, Holstebro, Denmark; 2 Department of Clinical Medicine, Aarhus University, Aarhus C, Denmark; 3 University Clinic for Hand, Hip and Knee surgery, Holstebro Regional Hospital, Holstebro, Denmark; 4 Department of Orthopaedic Surgery, Aarhus University Hospital, Aarhus C, Denmark; 5 Centre of Research in Rehabilitation (CORIR), Department of Clinical Medicine, Aarhus University, Aarhus C, Denmark

**Keywords:** Osteoarthritis, Cognitive-behavioral therapy, Pain Catastrophizing, Pain, Coping skills, Total knee arthroplasty

## Abstract

**Background:**

Total Knee Arthroplasty (TKA) is a common and generally effective procedure performed mainly due to advanced osteoarthritis, pain, physical disability and reduced quality of life. However, approximately 20% of the patients respond poorly to the surgery and chronic pain and disability following TKA remains a major health burden for many patients. Among the most well documented and powerful psychological predictors of poor outcome following TKA is pain catastrophizing. Recent research has shown that patients with these thoughts are at higher risk of having persistent pain and lower physical function after the operation than patients with low levels of pain catastrophizing before TKA. There is high need of developing treatments aimed at improving self-management for this group of patients and the aim of this study is to investigate the effectiveness of a patient education in pain coping on physical function and pain among patients with high pain catastrophizing score before a TKA.

**Methods:**

This study is a two-arm parallel group trial design including 56 patients with high levels of pain catastrophizing referred for total knee arthroplasty due to osteoarthritis. Patients eligible for participation will be randomized into the two arms, usual care or usual care and patient education. Usual care consists of operation and standard rehabilitation. The patient education consists of 7 individual sessions focusing on pain behavior and pain coping managed by a physiotherapist. Three before the operation and four after. Measurements will be taken at baseline before the operation and 3 and 12 months after the operation. Primary outcome will be pain after 12 months measured with VAS (Visual Analogue Scale). Secondary outcomes include physical function and activity, quality of life, pain management and psychological factors.

**Discussion:**

Only few studies have evaluated the effectiveness of psychological interventions on patients with high levels of pain catastrophizing before the operation. This trial will provide evidence for the effectiveness of a cognitive-behavioral patient education delivered by physiotherapists and may provide better functional outcome and less pain for a vulnerable group of TKA patients. We expect that the results can provide important new knowledge to the current care recommendations.

**Trial registration:**

Clinical Trials (NCT02587429). Registered 23 October 2015

## Background

Total Knee Arthroplasty (TKA) is a common and generally effective procedure and most of the operated patients have less pain and higher functional ability after the surgery [1]. However, some patients respond poorly to the surgery and chronic pain following TKA remains a major health burden for many patients [2]. “Poor” outcome following TKA is due mainly to disabling pain and impaired function [3, 4]. Thus, persistent knee pain was reported 12 months after TKA in 16.4% of the patients by Baker et al. who followed 9417 patient’ pain using the Oxford Knee Score (OKS) [3]. Similar pain results were reported by Nilsdotter et al. [5]. Most recently, in a systematic review Beswick and colleagues stated that 20% reported persistent function-limiting pain 6 months or more following a TKA, despite an apparently normally functioning prosthesis [4]. Routinely screening for patients at high risk for persistent pain after the surgery is suboptimal and not common practice [6]. This practice has potential to change because resent research shows that we are able to identify several psychological predictors of poor outcomes after knee arthroplasty. From the literature on non-surgical chronic pain the evidence suggests that pain disability does not result solely from the severity of pain but is largely influenced by patients’ adjustment and interpretation to their pain [7, 8]. Among the most well documented and powerful psychological predictors of poor outcome following TKA is pain catastrophizing [9]. Pain catastrophic thoughts are related to pain rumination, pain magnification and helplessness in the face of pain. New research has shown that patients with these thoughts are at higher risk of having persistent pain and lower physical function after the operation than patients with low levels of pain catastrophizing before TKA [2, 10–14].

**Fig. 1:**
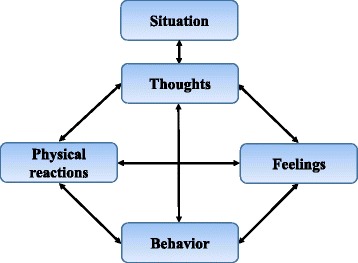
The cognitive triangle – The link between thoughts, feelings, physical reactions and behavior

Cognitive Behavioural Therapy (CBT) is one of the most widely accepted models in the field of pain psychology and the biopsychosocial approach of CBT focuses on the complex interaction between psychological (mood, personality, behavior, cognition etc.), social (cultural, familial, socioeconomic, medical, etc.) and biological (genetic, biochemical, etc.) factors. The biopsychosocial model assumes that health problems are hardly ever limited to just one domain of human experience but by multiple domains of human experience. CBT is a treatment that helps patients understand the thoughts and feelings that influence behaviors. CBT consists of a cognitive aspect and a behavioural aspect. The cognitive aspect is based on Becks cognitive model [15]: the way that individuals perceive a situation is more closely connected to their reaction than the situation itself. A person’s cognition has impact on their mood and emotions, their bodily reactions and their behavior. This is illustrated in Fig. 1. The second part of cognitive behavior therapy focuses on the actual behaviors that are contributing to the problem. The client begins to learn and practice new skills and one of the aims is to decrease maladaptive pain behaviors. The goal of CBT is to identify maladaptive thoughts and consequently change them into more realistic and constructive thoughts to modify feelings and behaviour and thereby the experience of pain [15, 16]. CBT protocols are typically delivered by psychologists but they are often not integrated into primary care where most of the patients are seen. It may be beneficial to use a single health care professional to deliver the patient education in pain coping and in connection with the operation where the patients meet the physical therapist several times. Potential advantages of using only one health care person may be a reduced overall cost to the health care system, increased opportunity of receiving the treatment and an experience of a more consecutive treatment because the patient has contact to fewer health care persons. Physical therapists have a history of teaching patients about pain and pain coping, and given their expertise with using physical treatments to treat patients pain and their experience in using the biopsychosocial approach in their treatment, physical therapists is a qualified choice to deliver a patient education in pain coping [17, 18].

Psychological interventions as CBT that train patients in pain coping produce significant reductions in pain and improvements in self-efficacy and psychological well-being in individuals with knee osteoarthritis (OA) [19–23]. However, there has been only been very few attempts to reduce pain by using a CBT approach in a population of patients with high preoperative pain catastrophizing score. In 2011 Riddle et al. published the results of a quasi-experimental study. They found that patients who had received training in pain coping skills had a greater reduction in pain and pain catastrophizing 2 months after TKA. Furthermore, they had a larger improvement in physical function measured with Western Ontario and McMaster Osteoarthritis Index (WOMAC) compared to patients in the control group [24]. On basis of this study Riddle et al. has published a study protocol describing a clinical randomized trial [25]. Since there have only been few studies evaluating the effect of psychological interventions on knee pain and no randomized controlled trials evaluating the effect on patients with high levels of pain catastrophizing, well designed randomized controlled trials to determine the effectiveness of such interventions are needed. Our study will supplement the trial from Riddle et al. but there will also be several differences i.e. the study from Riddle et al. will test a telephone-based pain coping skills training contrary to our study where the patients come to the hospital and meet a physiotherapist at all training sessions. Further, in our study a larger part of the sessions are placed preoperative instead of postoperative. There is need for studies examining how to improve care and treatment for patients with poor pain coping at high risk of chronic pain after the operation. Our study aims to target this need. Our primary aim is to investigate the effectiveness of a patient education in pain coping on pain among patients with high pain catastrophizing score before a TKA. The secondary aims of the study are to investigate the effectiveness on physical function, physical activity, quality of life and pain catastrophizing. We hypothesize that patients with high pain catastrophizing scheduled for TKA who participate in a patient education in pain coping will experience 1) lower pain intensity 2) a higher physical function 3) a higher self-reported quality of life 4) and a lower pain catastrophizing score after the operation than patients who do not participate in patient education.

## Methods/Design

### Design

This study is a single centre double-arm parallel group design trial with 1:1 allocation with 1-year follow-up. Measurements will be taken at baseline before the operation and 3 and 12 months after the operation.

### Participants and recruitment

We will recruit 56 patients from the Orthopedic outpatient clinic at Holstebro Regional Hospital.

To be eligible for inclusion the patients must fulfill the following criteria:

- Be able to speak, talk and understand Danish language.
- Provide informed consent.
- Have a primary diagnosis of osteoarthritis.
- Be scheduled for an elective unilateral total knee arthroplasty.
- Age ≥ 18 years.
- A score >22 on the Pain Catastrophizing Scale (PCS).

### Exclusion criteria

- Severe depression (at least three core symptoms and two accompanying symptoms) diagnosed with the Major Depression Inventory (MDI) [26].
- Scheduled for revision arthroplasty.
- Scheduled for a unicompartmental arthroplasty.
- Planning to have a contralateral knee arthroplasty within 1 year from the operation.
- Have a diagnosis of inflammatory arthritis.

**Fig. 2:**
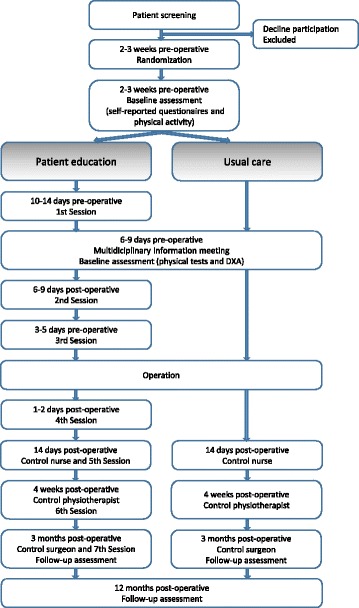
Diagram of the patient flow through the study

Patients eligible for participation will be randomized into the two arms, usual care or patient education (Fig. 2). The patients will not be aware of the study hypotheses and will not be informed about the association between a high PCS score and long-term pain after TKA. The randomization will take place approximately 2–3 weeks before the operation. Because the time between the patients are scheduled for operation by the surgeon and the operation is very short (2–3 weeks) the patients will be randomized immediately after they are included and before baseline assessments to be able to make three pre-operative appointments with the patients in the intervention group.

Randomization will be conducted by permuted blocks and an independent staff will prepare consecutively numbered, sealed envelopes. The physiotherapists and nurses who execute the baseline and follow-up testing will be blinded for the randomization. The patients and the three physiotherapists delivering the pain coping education cannot be blinded.

### Preoperative screening and selection of “high Pain Catastrophizing” patients

Patients will be screened preoperatively at the outpatient clinic after they are scheduled for a TKA using the questionnaire Pain Catastrophizing Scale (PCS) [27]. PCS consists of 13 questions where the patients rate their thoughts and feelings when they are experiencing pain. The total score is ranging from 0 indicating no “pain catastrophizing” to 52 indicating worst “pain catastrophizing”. Each item are rated on a 5-point scale ranging from “not at all” to “all the time”. The Danish version of PCS is considered valid for use in clinical pain samples and the internal consistency is found acceptable [28].

The cut-off score for PCS is chosen on basis of data collected in the years 2011–2013 at Holstebro Regional Hospital. 530 TKA patients answered the PCS before the operation. Median PCS score was 16 range (0–52). In the present trial we want to include the 33% of our TKA population with the highest PCS score. This means that we will use the 66th percentile of the total score to define the cut-off in this study. Thus, patients with a PCS score on 23 or higher are eligible for inclusion and in this study defined as patients with “high pain catastrophizing”.

### Control group

Patients in the control group will receive usual care. This consists of a multidisciplinary information meeting for patients and their relatives, approximately 1 week before the operation. After the operation the patients have contact with the hospital 4 times. Seven days (telephone call), 14 days, 4 weeks and 3 months after the operation they will be called in for a control visit with the nurse, physiotherapist and operating surgeon respectively. Further, some of the patients will receive physical rehabilitation in their local community.

### Intervention group

The intervention group will in addition to the described usual care participate in a patient education based on cognitive-behavioral therapy. We wanted to design a patient education that fits into a hospital setting with relatively few session contrary to previous studies were the CBT intervention most commonly range from 10 to 12 sessions [20, 23, 29]. Our patient education consists of seven individual sessions. Three sessions before the operation and four after. A similar trial has recently shown similar amounts of sessions to be effective [24]. Except session one and three all the sessions will be in relation to the above mentioned planned visits at the hospital. The first session will be delivered approximately 2 weeks before the operation and the last one 3 months after the operation. Each session will be approximately 45 min in length. The patient education will be delivered by one of three physiotherapists with several years of experience in treating patients with TKA and a special interest in the CBT area. The three physiotherapists have participated in a 3-day training program delivered by two psychologists specialized in cognitive behavioral therapy and pain management. The patient education is developed together with the two psychologists and the three physiotherapists. The intervention is pilot tested on 3 patients with high pain catastrophizing score before we started including patients.

The same physiotherapist will follow the patient through all the 7 sessions. To make sure that the physiotherapists will all deliver the intervention in the same way they will be observed by each other on a regular basis.

### Content

The three physiotherapists will follow a pre-specified manual containing the content of each of the seven sessions. Though the manual will leave some flexibility to discuss the patient’s individual needs and problems in each of the sessions. The content of the manual is inspired by Rolving et al. [30]. In the first session, the patient will be handed out a patient hand-book containing all the key points from the sessions and material for the homework. Further, they will receive an mp3 player with different relaxation and mindfulness exercises and a pain diary. The content of each of the seven sessions is described in Table 1.

**Table 1:** An overview of the content of each of the seven sessions in the cognitive-behavioral intervention

Session/time	Focus and skills
1. session	Introduction to the patient education.
2 weeks preoperative	Causes and consequences of pain. Different types of pain.
	Introduction to the cognitive triangle – The link between thoughts, feelings, bodily reactions and behavior.
	*Homework*: Identify and write down thoughts in relation to painful or stressful situations.
2. session	Active and passive coping strategies.
1 week preoperative - relatives are invited to participate	How to cope with pain and distress in relation to family, relatives and work.
	The consequences of fear avoidance and the link between activity and pain.
	Relaxation and mindfulness exercise.
	*Homework*: Identify and write down your own coping strategies when in pain or distress. Relaxation and mindfulness exercise.
3. session	Appropriate activity management – activity pacing.
3–5 days preoperative	Pleasant activity scheduling.
	Goal setting.
	Introduction to pain diary.
	*Homework*: Identify five activities you used to enjoy and would like to do again.
4. session	Summary of learned skills from previous sessions.
During hospitalization	Goal setting for the next 14 days.
1–2 days postoperative	Appropriate rest and activity.
	*Homework*: Use the pain diary the next 14. days.
5. session	The cognitive triangle – The link between thoughts, feelings, bodily reactions and behavior.
14 days postoperative	Learning how to change negative automatic thoughts and catastrophic pain-related thoughts into more realistic thoughts by using cognitive restructuring techniques
	Pleasant activity scheduling and activity pacing.
	*Homework*: Use pacing techniques and pleasant activity scheduling to restart daily activities and hobbies. Write down how it affects your mood and pain level.
	Identify and write down troubled thoughts and how they affect your feelings, bodily reactions and behavior. Consider alternative realistic thoughts.
6. session	Restructuring of inappropriate thoughts.
4 weeks postoperative	Working with the patient’s individual problems.
	Goal setting for the next 2 months.
	*Homework*: Identify catastrophic and negative thoughts and try to change them to alternative more realistic thoughts.
7. session	Brush up from the 6 previous sessions and a reflection of which coping techniques and cognitive techniques the patient can and will use in the future
3 months postoperative	How to manage and control flare-ups
	Plan for the future

All sessions begins with questions and a talk about the homework from the previous session

The patient education involves three main components: a) education about pain and the role pain coping skills can play in pain management, b) systematic training in a variety of cognitive and behavioral pain coping skills, c) training in how to apply learned coping skills to real life situations that are particularly challenging to the patient.

The goal of the patient education is to teach the patients about pain in order to gain a better understanding of how cognitions and behavior affect the pain experience. We want them to understand the link between thoughts, feelings, bodily reactions and behavior and to realize their own role in controlling their pain experience. The patients shall learn to use appropriate coping strategies such as activity pacing and pleasant activity scheduling to increase their activity level and observe the resultant impact on their pain related cognitions. Additionally, the patients will be trained in cognitive restructuring. Using techniques drawn from cognitive therapy the patients will be taught how to identify irrational, maladaptive and catastrophic related thoughts that contribute to pain and how to replace them with alternative rational and more positive thoughts. Each patient will learn about their own early warning signs of setbacks and potential high risk situations and how to use the coping skills learned in the seven sessions to deal with future challenges.

### Outcome assessment

The data presented in Table 2 will be measured before the operation and 3 and 12 months postoperative. Physical function and DXA scan will be measured during clinic visits. All the self-reported questionnaires are send by e-mail or post to the patients 2 weeks before the visit together with the tri-axial accelerometer. The primary outcome measure of the study is the 100-mm Visual Analogue scale (VAS), which is a self-reported pain measurement ranging from 0 indicating “no pain” to 100 indicating “worst pain imaginable” [31].

**Table 2:** Primary and secondary outcome measures

Measure	Instrument	Collection points and method
Primary outcome:		
Self-reported Pain intensity	Visual Analogue Scale (VAS)	Baseline, 3 and 12 months follow up by post/email
Secondary outcomes:		
Self-reported function	SF-36 Physical Function	Baseline, 3 and 12 months follow up by post/email
Self-reported function and pain	Oxford Knee Score (OKS)	Baseline, 3 and 12 months follow up by post/email
Self-reported health status	EQ-5D-3 L	Baseline, 3 and 12 months follow up by post/email
Self-reported pain catastrophizing	Pain Catastrophizing Scale (PCS)	Baseline, 3 and 12 months follow up by post/email
Self-reported daily function and quality of life	Knee injury and Osteoarthritis Score (KOOS)	Baseline, 3 and 12 months follow up by post/email
Self-reported pain self-efficacy	Pain Self-Efficacy Questionnaire (PSEQ)	Baseline, 3 and 12 months follow up by post/email
Daily activity	Tri-axial accelerometer	Baseline, 3 and 12 months follow up by post/email
Physical function	6 min’ walk test (6MWT)	Baseline, 3 and 12 months follow up test in clinic
Physical function	Sit to stand 30 s. (STS30)	Baseline, 3 and 12 months follow up test in clinic
Muscle mass and bone mineral density	DXA scan	Baseline, 3 and 12 months follow up scan in clinic
Other measures		
Healthcare visits	Self-report (log book)	0–3 months’ post-operative at home
Smoking	Self-reported questionnaire	Baseline, 3 and 12 months follow up by post/email
Pain killers	Self-reported questionnaire	Baseline, 3 and 12 months follow up by post/email
Alcohol	Self-reported questionnaire	Baseline, 3 and 12 months follow up by post/email
Education	Self-reported questionnaire	Baseline, 3 and 12 months follow up by post/email
Social status	Self-reported questionnaire	Baseline, 3 and 12 months follow up by post/email

Secondary outcomes will be several self-reported questionnaires: SF-36 (physical function) [32], Oxford Knee Score (OKS) [33], EQ-5D [34], Pain Catastrophizing Scale (PCS) [28], Knee injury and Osteoarthritis Score (KOOS) [35] and Pain Self-Efficacy Questionnaire (PSEQ) [36–38]. Daily activity will be measured over 7 days with tri-axial accelerometers [39, 40]. Two performance-based measures, the 6-minute walk test [41, 42] and sit to stand on 30 s [43–45] will be assessed to measure Physical function. The performance tests are supervised by a trained staff following a SOP to insure standardization. Muscle mass and bone mineral density (BMD) will be measured with a DXA scan and the patients are asked to record health care visits after the operation in a logbook. Other measures collected with self-reported questionnaires are smoking, alcohol, work, education, use of pain killers and social status. Further information about comorbidity, operation, complications, rehabilitation and previous knee surgery will be registered by medical records.

### Sample size and statistical analysis

The primary outcome measure will be VAS under activity measured on a continuous 100 mm scale 12 month after the operation. Clinically important differences between the two groups is detected to be 18 mm [46, 47]. Based on earlier studies we assume a common standard deviation between participants of 19 mm on VAS [2, 10, 48]. With a significance level at 0.05 and the power at 0.90 the study needs 23 patients in each arm. Taking into account a loss to follow-up after 1 year on approximately 20% we aim at including 28 patients in each group a total of 56 patients.

Clinical and demographic characteristics as well as baseline data will be presented to show the baseline comparability of the two groups. Data from the patients who withdraw from the study will also be examined. For each group descriptive statistics will be presented at baseline and three and 12 months postoperative. Normally distributed data are described by means and standard deviation (SD), and data not normally distributed by medians and interquartile range (IQR). The primary analysis will use the intention-to treat principle including all randomised participants. Patients who discontinue the intervention will be encouraged to participate in the follow up test anyway, and those who accept will be included in the analyses according to their original group assignment. The primary outcome pain is measured on a continuous scale and between-group mean differences and 95% confidence intervals from baseline to 3 and 12 months will be analyzed by a mixed effects linear regression model with a random person level and systematic effects of time, group and the interaction between time and group. We will employ multiple imputation methods in order to assess the sensitivity of the mixed model to any informative missing values. Imputations will be performed using baseline characteristics and non-missing pain measurements on individual levels. Secondary outcomes will be analysed as above for normally distributed measures or for binary outcomes, we will use logistic regression with a robust variance estimate to account for the repeated measurements. Standard diagnostic plots will check model assumptions. The statistical analyses will be performed using STATA 13 (StataCorp, College Station, TX) software package. The significance level will be set at 0.05.

### Ethical considerations

The study is approved by The Central Denmark Region Committee on Biomedical Research Ethics (journal no. 1-10-72-64-15) and The Danish Data Protection Agency and is registered at www.clinicalTrials.gov (NCT02587429). The study will be conducted in accordance with the Good Clinical Practice Guidelines and the Declaration of Helsinki. All patients are ensured minimum standard treatment and the patient education carries no risk to the patients.

## Discussion

Knee OA is one of the most common forms of arthritis and an increasing proportion of older people have to live with chronic diseases in the future. This will carry a major economic burden for the healthcare system and recent research shows that there is high need of developing treatments aimed at improving self-management for a selected group of patients at high risk of having long-term pain and physical disability after a TKA. CBT interventions are traditionally delivered by psychologists and using physiotherapists to deliver psychological interventions is fairly novel. However, physiotherapists have a history of teaching patients about pain and pain coping. Further, they normally see the patients several times in connection with the operation and taken into account the high number of TKA operations and the limited availability of psychologists at the hospital physiotherapists are highly qualified to provide this patient education. Recent studies shows that both nurses and physiotherapists are sufficient to deliver high standard pain coping skills training [17, 49].

This study design has several strengths. First, the patients are screened on basis of their risk of having chronic pain after the operation. Only patients with high levels of pain catastrophizing will be eligible for the patient education and the education will be targeted these patients. Second, the outcome measures are valid and reliable, and include a range of both self-reports and objective measures. Third, there has only been few studies evaluating the effect of psychological interventions on knee OA pain and no RCT’s evaluating the effect on patients with high levels of pain catastrophizing. Thus, good RCT’s are needed to determine the effectiveness.

There are some limitations as well. First, due to the different amount of attention paid to the patients in the two groups, a Hawthorne effect is possible [50, 51]. However, all patients will receive at least standard care which includes several contacts to both nurses, physiotherapists and doctors. Second, DXA scan, 30 s sit-to-stand and 6 MWT will be conducted after the first session in the intervention group. This procedure is chosen to be able to maintain the blinding of the physiotherapists by testing all the patients in the two groups the same day. However, the content of the first session is mostly introduction and since these outcome measures are not patient reported we do not think this will have an impact upon the results. Third, the patients will be randomized before baseline assessment. The reason for this is that the time between the patients are referred to a TKA and the operation is short and to be able to see the patients in the intervention group three times before the date for the operation this procedure is chosen. However, none of the patients are informed about the association between a high PCS score and long-term pain after TKA, so we don’t think this will affect the results.

We consider the external validity of the trial to be high while the treatment of TKA patients is almost similar all over the country. This makes it easier to implement at other Hospitals.

This project is expected to improve the overall treatment effect for a group of patients we today do not know how to treat the best way. We hope that this study can provide better functional outcome and less pain for the patients after the operation and that the study can produce new knowledge and inspire similar developments of interventions to other diseases.
